# Special Issue: The Role of Genetics in Dementia

**DOI:** 10.3390/ijms26178355

**Published:** 2025-08-28

**Authors:** Ioannis Liampas

**Affiliations:** Department of Neurology, Faculty of Medicine, School of Medicine, University of Thessaly, Mezourlo Hill, 41100 Larissa, Greece; iliampas@uth.gr or liampasioannes@gmail.com

In recent years, the integration of genetic and epigenetic insights has significantly enriched our understanding of dementia’s complex neurobiology. Based on these findings, research is steadily moving towards precision diagnostics and personalized therapeutics. However, our knowledge in this field is still evolving, with much more to unravel.

Monogenic forms of dementia are caused by rare mutations in single loci, most notably APP (β-amyloid precursor protein), PSEN1 (presenilin 1), and PSEN2 (presenilin 2) in Alzheimer’s disease (AD) [1] MAPT (microtubule-associated protein tau), GRN (granulin precursor), and C9orf72 (chromosome 9 open reading frame 72) in frontotemporal dementia (FTD) [2]. These mutations typically lead to early-onset (EO) disease and follow Mendelian inheritance patterns. While they represent only a small proportion of dementia cases, monogenic forms offer important insights into disease mechanisms like β-amyloid aggregation or TAR DNA-binding protein 43 (TDP-43) pathology.

Most cases of late-onset (LO) dementia are polygenic, influenced by many common genetic variants, each contributing a small risk. Polygenic risk scores (PRSs) compound the individual effects of these variants into a single metric to estimate an individual’s genetic predisposition to dementia [3]. PRSs may be applied to stratify risk in asymptomatic individuals [4] and enhance predictive models paired with clinical or imaging data [5]. *Apolipoprotein E4* (*APOE4*) carriage remains the strongest individual genetic risk factor for LOAD, but PRSs can capture broader, additive risk [6].

Although genome-wide association studies (GWAS) usually focus on individual variants and their additive effects, in reality, genes interact in networks [7]. Studies focusing on epistasis (gene-gene interactions) have captured part of the “missing” heritability in dementia and have explained several inconsistencies of published research [8]. These findings may lead to better disease modeling and the identification of new pathophysiological pathways.

Epigenetic mechanisms (such as DNA methylation) [9], gene–environment interactions (especially with respect to *APOE*) [10], and pharmacogenomics [11] have also attracted increasing attention in dementia research. Apart from superior personalized risk assessments [12], these genetic aspects may also enable clinicians to apply tailored prevention strategies and inform therapeutic decision-making [13].

This Special Issue aimed to capture advances in this promising field of neurogenetics and contribute to pushing dementia research into new territory, towards early and accurate detection, refined classification, and personalized interventions. Case reports, reviews, regional genomic studies, and transcriptome-wide association analyses that report novel mutations, gene–gene interactions, regional genetic divergence, polygenic influences, and genotypic–phenotypic associations are included. These insights enhance our understanding of major neurocognitive entities and pave the way to personalized medicine.

One study reported a case of an individual with EOAD, harboring the *PSEN1* Glu318Gly variant along with additional potential risk mutations, specifically *SORL1* (sortilin-related receptor L) Glu270Lys, *ABCA7* (ATP binding cassette sub-family A member 7) Val1946Met, *TOMM40* (translocase of outer mitochondrial membrane 40) Arg239Trp, and *GRN* Ala505Gly. The authors focused on potential gene interactions, i.e., *PSEN1* Glu318Gly may not be a causative mutation for EOAD on its own, but it may interact with other potential risk variants. These findings deepen our understanding of the genetic heterogeneity and complexity of EOAD [14].

Another case study described a Korean patient with familial EOAD with a novel *PSEN1* His214Asn mutation. This study emphasized the role of this mutation in compromising the structural stability of *PSEN1*, altering γ-secretase cleavage dynamics and ultimately resulting in elevated amyloid production [15].

Another study examined the regional differences in genetic mutations associated with AD and frontotemporal dementia (FTD) across Italy’s geographic regions [16]. It highlights notable mutational variability, suggesting regional genetic divergence in dementia predisposition and subsequent phenotypic heterogeneity.

A narrative review explored the role of the *APOE* gene in α-synucleinopathies [17]. It linked *APOE4* to dementia with Lewy bodies (DLB) and Parkinson’s disease dementia (PDD). Of note, in individuals with DLB, *APOE4* carriage appears to be intermediately prevalent between AD and PDD (AD > DLB > PDD > PD). Multiple system atrophy (MSA) was not associated with *APOE* genotypes and PD presented more complex associations, modified by ethnicity. These findings delve into the complex interactions between *APOE* and different neurodegenerative pathologies that may lead to cases with mixed (AD and non-AD) phenomenology.

An updated review investigated genetic factors underlying mild behavioral impairment (MBI), which may serve as a precursor to neurocognitive entities, and summarized current genetic associations and mechanisms [18]. The *APOE* genotype and *MS4A* locus are associated with affective dysregulation, *ZCWPW1* with social inappropriateness and psychosis, *BIN1* and *EPHA1* with psychosis, and *NME8* with apathy. These findings, along with the results from studies exploring polygenic risk scores, offer insights into the potential underlying neurobiological mechanisms of neuropsychiatric symptoms.

One study conducted transcriptome-wide association analyses across multiple ancestries, connecting gene expression patterns with cognitive performance, white-matter hyperintensity burden, and Alzheimer’s disease susceptibility [19]. Many of the identified genes were in pathways related to innate immunity, vascular dysfunction, and neuroinflammation. These findings offer neurobiological insights into common and ancestry-specific transcriptomic signals linked to neurocognitive entities.

Taken together, the aforementioned findings shed additional light on the labyrinthine genetic architecture of major neurocognitive entities. Future research should confirm and capitalize on these findings. The integration of multi-omics (genomics, epigenomics, proteomics, metabolomics, and so on) in dementia may help us to understand the complex neurobiology of dementia holistically and in depth and identify very early molecular signatures of the disease [20].
